# Irradiation in Adulthood as a New Model of Schizophrenia

**DOI:** 10.1371/journal.pone.0002283

**Published:** 2008-05-28

**Authors:** Yasuhide Iwata, Katsuaki Suzuki, Tomoyasu Wakuda, Norihito Seki, Ismail Thanseem, Hideo Matsuzaki, Takayoshi Mamiya, Takatoshi Ueki, Sumiko Mikawa, Takeshi Sasaki, Shiro Suda, Shigeyuki Yamamoto, Kenji J. Tsuchiya, Genichi Sugihara, Kazuhiko Nakamura, Kohji Sato, Nori Takei, Kenji Hashimoto, Norio Mori

**Affiliations:** 1 Department of Psychiatry and Neurology, Hamamatsu University School of Medicine, Shizuoka, Japan; 2 Department of Anatomy, Hamamatsu University School of Medicine, Shizuoka, Japan; 3 Mental Development Research Center, Hamamatsu University School of Medicine, Shizuoka, Japan; 4 Mental Development Research Center, Osaka University Graduate School of Medicine, Osaka, Japan; 5 Department of Chemical Pharmacology, Faculty of Pharmaceutical Sciences, Meijo University, Nagoya, Japan; 6 Division of Clinical Neuroscience, Chiba University Center for Forensic Mental Health, Chiba, Japan; James Cook University, Australia

## Abstract

**Background:**

Epidemiological studies suggest that radiation exposure may be a potential risk factor for schizophrenia in adult humans. Here, we investigated whether adult irradiation in rats caused behavioral abnormalities relevant to schizophrenia.

**Methodology/Principal Findings:**

A total dose of 15-Gy irradiation in six fractionations during 3 weeks was exposed to the forebrain including the subventricular zone (SVZ) and subgranular zone (SGZ) with male rats in the prone position. Behavioral, immunohistochemical, and neurochemical studies were performed three months after fractionated ionizing irradiation. Three months after fractionated ionizing irradiation, the total numbers of BrdU-positive cells in both the SVZ and SGZ zones of irradiated rats were significantly lower than those of control (sham-irradiated) rats. Hyperactivity after administration of the dopaminergic agonist methamphetamine, but not the N-methyl-D-aspartate (NMDA) receptor antagonist dizocilpine, was significantly enhanced in the irradiated rats although spontaneous locomotion in the irradiated rats was significantly lower than that of controls. Behavioral abnormalities including auditory sensory gating deficits, social interaction deficits, and working memory deficits were observed in the irradiated rats.

**Conclusion/Significance:**

The present study suggests that irradiation in adulthood caused behavioral abnormalities relevant to schizophrenia, and that reduction of adult neurogenesis by irradiation may be associated with schizophrenia-like behaviors in rats.

## Introduction

Schizophrenia is a heterogeneous and multifactorial disease with complex interactions between genetic liability and environmental factors. A number of epidemiological studies have proposed perinatal events with potential harmful neurodevelopmental impacts as major environmental risk factors [1]–[4], but few studies have revealed risk factors in adulthood. Interestingly, some epidemiological studies suggest that exposure to ionizing radiation may be a risk factor for schizophrenia in adult humans [5]. First, a higher prevalence (6%) for schizophrenia was reported in the atomic bomb survivors in Nagasaki, Japan [6]. Second, four years after the Chernobyl accident in 1986, the incidence of schizophrenia in the exclusion zone was significantly higher than that in the general population (5.4 per 10,000 in the exclusion zone versus 1.1 per 10,000 in the Ukraine in 1990) [7]. Third, the incidence for schizophrenia was shown to be high in people living in the region of the Semipalatinsk nuclear weapon testing area in Kazakhstan: 29% of all registered mental patients residing in the area were suffering from schizophrenia and among those, 42.3% were born before the first nuclear test explosions [5]. Furthermore, the incidence for schizophrenia has also been shown to be high in rural areas in India that have high natural background radiation [5]. Taken together, the findings suggest that ionizing radiation may be an environmental trigger that can actualize a predisposition to schizophrenia or indeed cause schizophrenia-like disorders [5].

In both pediatric and adult patients, cranial radiation therapy causes debilitating cognitive deficits that are poorly understood [8]. However, accumulating evidence suggests that radiation-induced cognitive deficits in animals may be associated with a decrease in hippocampal proliferation and a decrease in adult neurogenesis [9]–[15]. Interestingly, Reif et al. [16] reported a reduction in the proliferation of hippocampal neural stem cells in the postmortem brains of schizophrenic patients. Therefore, it is likely that adult neurogenesis plays an important role in the pathophysiology of psychiatric diseases including schizophrenia [17]. Given the role of neurogenesis in radiation-induced cognitive deficits, we hypothesized that reduction of adult neurogenesis by irradiation may be implicated in the pathophysiology of schizophrenia in adulthood. The present study was, therefore, undertaken to examine whether irradiation in adult rats causes behavioral abnormalities relevant to schizophrenia.

## Methods

### Animals

Adult male Sprague-Dawley rats (Japan SLC, Hamamatsu, Japan), aged 8 weeks and weighing 280–300 g, were housed in groups of three animals per cage under standard conditions (22±0.5°C, 12∶12 light-dark cycle, lights on at 7:00 AM). All procedures were approved by the Guide for Animal Experimentation of the Hamamatsu University School of Medicine and Chiba University Graduate School of Medicine. Irradiated group and control group were 148 and 144 rats, respectively. All analyses were performed three months after the last irradiation. Six rats from both groups were used for neurotransmitter quantification, and eight rats were used for cell counting. Nocturnal activity, methamphetamine-treated response, and dizocilpine-treated response were measured using 17, 17 and 18 pairs of rats, respectively. Thirty-five irradiated rats and 33 control rats were used for cognitive function tests of social interaction (6 rats each), eight-arm radial maze (17 irradiated and 15 control rats), and Morris water maze (12 rats each). Prepulse inhibition (PPI) test was analysed in 23 irradiated and 21 control rats. Twelve rats for each group were used for analysis of clozapine effect on PPI deficits.

### Fractionated ionizing irradiation

The irradiation was done with a Stabilipan 2 (Siemens) therapeutic unit (150 kV and 20 mA). A total dose of 15-Gy irradiation in six fractionations during 3 weeks was exposed to the forebrain including the subventricular zone (SVZ) and subgranular zone (SGZ) with rats in the prone position. The other parts of the head and whole body were protected by a lead shield. Sham-irradiation controls underwent the same procedures as the experimental animals, but did not receive irradiation.

### Immunohistochemistry and stereological analysis

Twenty-four hours after intraperitoneal injection of BrdU (100 mg/kg; Sigma-Aldrich Japan Inc., Tokyo, Japan), brains were fixed with 4% paraformaldehyde. They were coronally sectioned at 30 µm, and eight-section series were collected. Serial sections were stained with mouse monoclonal anti-BrdU antibody (0.6 µg/mL; Becton Dickinson Immunocytometry Systems, CA, USA) and biotinylated horse-anti-mouse IgG (1∶160; Vector Lab. Inc., CA, USA). The signal was visualized using an ABC kit (Vector Lab. Inc., CA, USA) and 3, 3′-diaminobenzidine (Sigma- Aldrich Japan Inc., Tokyo, Japan). Other series were stained with Cresyl Violet for counting granule cells. The numbers of BrdU-labeled nuclei in the SVZ and SGZ of the dentate gyrus, and granule cells in the dentate gyrus were evaluated with Stereo Investigator (version 6, MicroBrightField Japan, Inc., Chiba, Japan). The SVZ estimates were made from two sections each anterior and posterior to the decussation of the corpus callosum (Bregma 1.60 mm). SGZ and granule cell layer estimates were made from an 8-section series between the top and end of the hippocampus. The volumes of each portion were estimated using Cavalieri's principle [18].

### Measurement of dopamine, DOPAC and amino acids

Dopamine and its major metabolite DOPAC in rat brain sample were measured by high-performance liquid chromatography (HPLC) coupled with electrochemical detection (Eicom Co., Ltd., Kyoto, Japan) as reported previously [19]. Amino acids (glutamine, glycine, glutamate, D-serine, L-serine) in rat brain samples were measured by column-switching HPLC (Shimadzu Co., Ltd., Kyoto, Japan) as reported previously [20].

### Psychostimulant-induced hyperlocomotion

Spontaneous nocturnal locomotor activity was measured for six hours in the middle of dark phase (21:00–3:00). Abnormalities in dopaminergic neurotransmission were tested by hyperlocomotion induced by the psychostimulant methamphetamine. Locomotor activity was monitored under an infrared ray passive sensor system (SCANET-SV20, Melquest Ltd., Toyama, Japan). After a 30-minute acclimation period, rats were intraperitonealy (i.p.) injected with methamphetamine (2.0 mg/kg, Dainippon Pharmaceuticals Ltd, Osaka, Japan) or dizocilpine ((+)-MK-801; 0.03 mg/kg, Sigma-Aldrich, St Louis, MO), and horizontal locomotor activities were measured for 2 hours.

### Social interaction

Social interaction was tested in a wooden arena (90×90×30 cm high) placed in a dimly lit room. Each rat was tested for 10 min with a weight-matched partner that had a similar treatment condition but was from a different home cage. Social interaction was assessed by the time spent interacting, including sniffing, following, crawling over or under, grooming, and aggressive behaviors.

### Eight-arm radial maze

Spatial working memory was analyzed with an automated eight-arm radial maze system in a manner similar to that described previously [21]. Rats were placed on the central platform and allowed to get all eight pellets within 10 min. The rats went through 1 trial per day. When a rat could take seven pellets within 1 error for five consecutive days, the rat was administrated 10 daily sessions for working memory assessment. A 30-sec delay was initiated after four pellets had been taken by confining the rats in the center with a shutter. After opening the shutter, the rat was allowed to get the remaining 4 pellets. The number of revisits to arms from which pellets had already been taken was used as the working memory error. Data acquisition and control of shutter were performed using Image RM 2.00 (O'Hara & Co., Ltd. Tokyo, Japan), modified NIH Image program (available at http://rsb.info.nih.gov/nih-image/).

### Morris water maze

Spatial reference memory was assessed using the Morris water maze (180 cm in diameter circular pool). A submerged translucent platform was fixed in the center of a quadrant (north). Training sessions consisted of placing the rat into the water maze at one of three randomly chosen start positions (south, east, west) and allowing it to swim to the platform for 60 sec. On the next day, after rats were trained for 5 days with four trials per day, the platform was moved to the opposite quadrant (south). A probe trial was carried out after four trials identical to the training sessions. The platform was removed and rats were allowed to swim freely for 60 sec. The time spent in the quadrant where the platform has been previously located was used as an index of spatial reference memory.

### Prepulse Inhibition of acoustic startle response

The rats were tested for their acoustic startle responses (ASR) in a startle chamber (SR-LAB, San Diego Instruments, CA, USA). The sessions consisted of five trial types: 1) pulse alone, a 40-millisecond broadband burst; 100 milliseconds preceding the pulse, a 20-millisecond prepulse (PP) that was either 2) 4 dB (PP74), 3) 8 dB (PP78), or 4) 16 dB (PP86) over the background (70 dB), and 5) background only (no stimulus). The amount of prepulse inhibition (PPI) is expressed as the percentage decrease in the amplitude of the startle response caused by presentation of the prepulse (%PPI).

To examine the effects of clozapine on PPI deficits in irradiated rats, vehicle (0.8% acetic acids; 1 ml/kg for 3 weeks) or clozapine (5 mg/kg/day for 3 weeks) were i.p. administered into control and irradiated rats (control/vehicle = 6, control/clozapine = 6, irradiated/vehicle = 6, irradiated/clozapine = 6). After the chronic (3 weeks) administration of vehicle or clozapine, PPI of acoustic startle response was examined as described above.

### Statistical analysis

Data are expressed as means±standard errors of the means (SEM). The data from two experimental groups were compared by unpaired t-test, except for PPI analysis, which was performed by a two-way (irradiation and prepulse intensity) analysis of variance (ANOVA). The level of significance was set at *p*<0.05.

## Results

**Figure 1:**
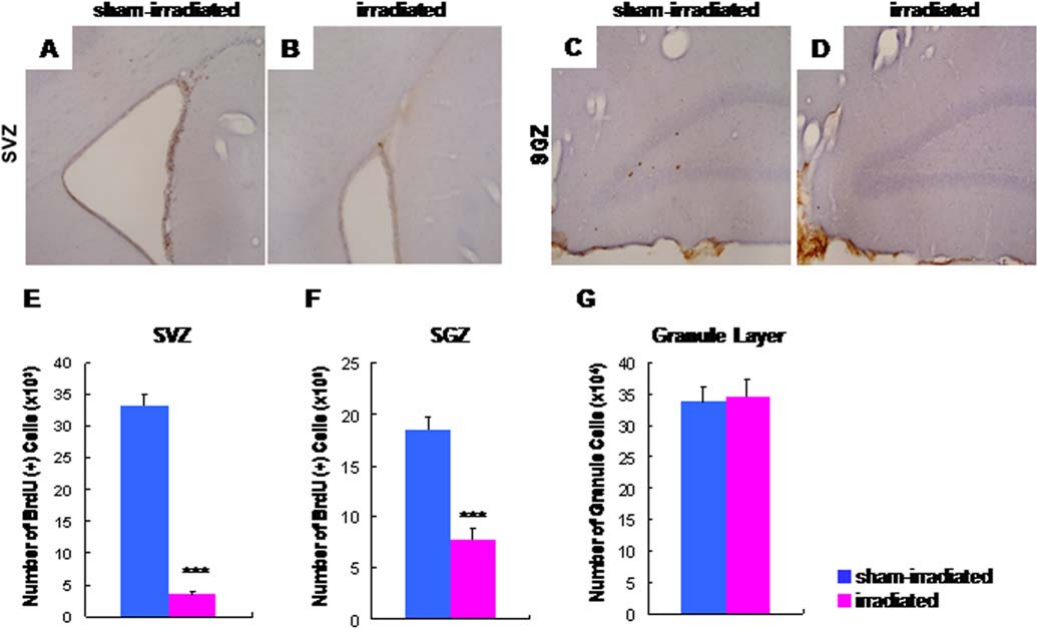
Decreased neurogenesis in the irradiated adult rats. The BrdU-positive cells in both SVZ (B and E) and SGZ (D and F) of the irradiated rats (n = 8) were significantly fewer than those (SVZ, A and E; SGZ, C and F) of control (sham-irradiated) rats (n = 8). Data are given as means±SEM. ***p<0.001 as compared with controls. (G) The total numbers of granule cells in the dentate gyrus in irradiated rats (n = 6) and control (sham-irradiated) rats (n = 6) were not different.

Three months after fractionated ionizing irradiation, the total numbers of BrdU-positive cells in both the subventricular (SVZ: **Figure 1B**) and subgranular (SGZ: **Figure 1D**) zones of irradiated rats were significantly lower than those (SVZ: **Figure 1A**, SGZ: **Figure 1C**) of control (sham-irradiated) rats (SVZ: **Figure 1E**, SGZ: **Figure 1F**). These findings are consistent with those of previous reports [9], [10], [12]. In contrast, the cumulative numbers of granule cells in the granule layer were not different between the two groups (**Figure 1G**).

**Figure 2:**
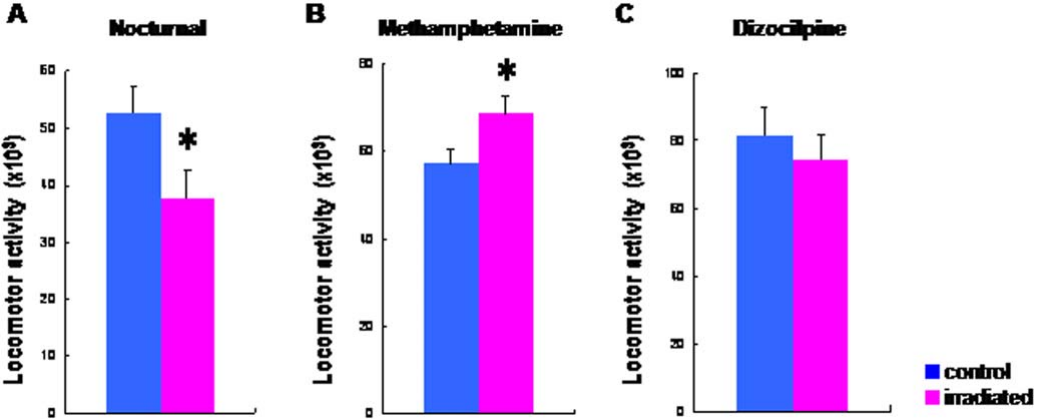
Spontaneous locomotion and response to methamphetamine and dizocilpine in irradiated adult rats. (A) Nocturnal spontaneous locomotion (21:00–3:00) in the irradiated rats (n = 17) was significantly lower than that of control (sham-irradiated) rats (n = 17). (B) Horizontal locomotor activity during the 120-min period after administration of the psychostimulant drug methamphetamine (2 mg/kg, i.p.) in irradiated rats (n = 17) was significantly higher than that of control rats (n = 17). (C) Horizontal locomotor activity during the 120-min period after administration of dizocilpine (0.03 mg/kg, i.p.) in the irradiated rats (n = 18) was not different from that of control rats (n = 18). Data are given as means±SEM.

**Figure 3:**
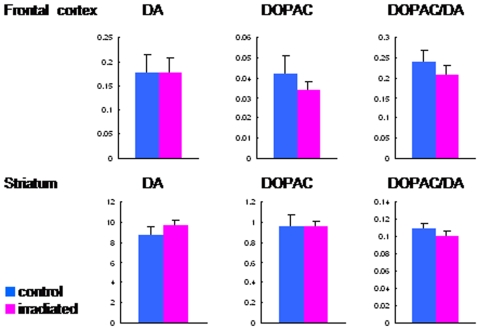
Dopamine and its major metabolite DOPAC levels in the frontal cortex and striatum of rat brain. Levels of dopamine and its major metabolite DOPAC, and dopamine turnover (DOPAC/dopamine ratio) in the frontal cortex and striatum were determined by HPLC analysis. There are no differences between irradiated rats (n = 6) and sham-control rats (n = 6).

**Figure 4:**
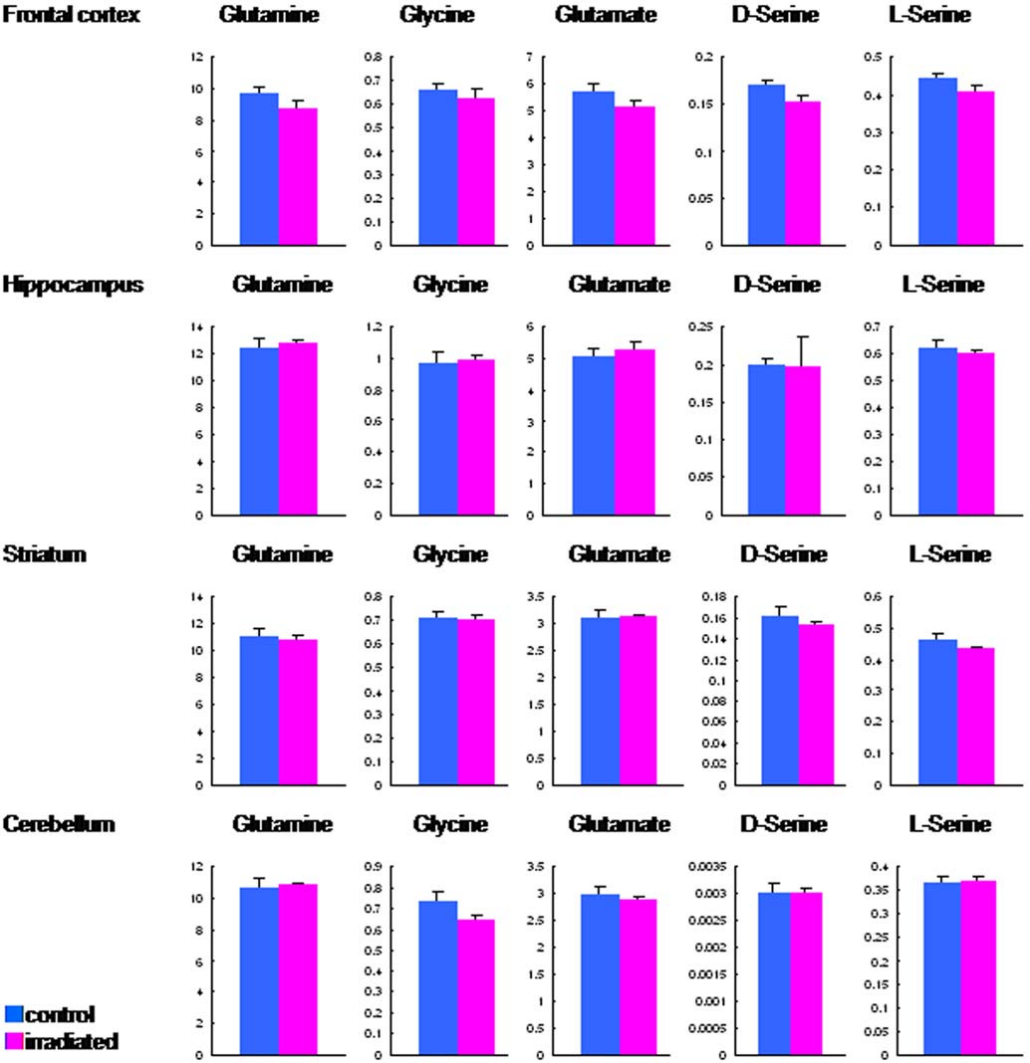
Levels of amino acids in the brain. Levels of amino acids (glutamate, glycine, glutamine, D-serine, L-serine) related with the NMDA receptor neurotransmission in the frontal cortex, hippocampus, and striatum, and cerebellum were determined by HPLC analysis. There are no differences between irradiated rats (n = 6) and sham-control rats (n = 6).

As shown in **Figure 2A**, the nocturnal spontaneous locomotion of irradiated rats was significantly (t = 2.34, df = 38.1, p = 0.025) lower than that of control rats. Furthermore, locomotor activity after administration of methamphetamine (2.0 mg/kg, i.p.) to irradiated rats was significantly (t = −2.26, df = 32, p = 0.031) higher than that of control (sham-irradiated) rats (**Figure 2B**). HPLC analysis revealed that levels of dopamine and its major metabolite DOPAC, and dopamine turnover (DOPAC/dopamine ratio) in the frontal cortex and striatum of irradiated rats were not different from those of sham-control rats (**Figure 3**). In contrast, locomotor activity after administration of the NMDA receptor antagonist dizocilpine ((+)-MK-801, 0.03 mg/kg, i.p.) to irradiated rats was not different from that of sham-control rats (**Figure 2C**). Furthermore, levels of amino acids (glutamate, glycine, glutamine, D-serine, L-serine) related with the NMDA receptor neurotransmission in the frontal cortex, hippocampus, and striatum, and cerebellum of irradiated rats were not different from those of sham-control rats (**Figure 4**).

**Figure 5:**
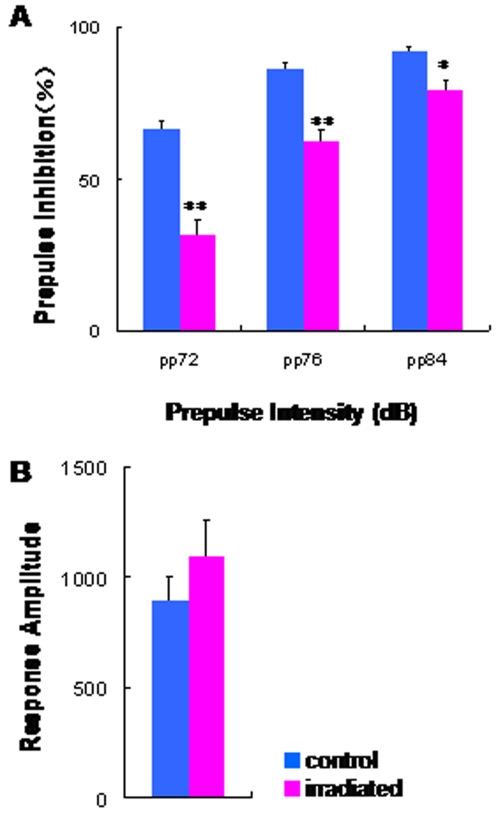
Sensorimotor gating deficits in the irradiated adult rats. (A) Auditory sensorimotor gating test: The irradiated rats (n = 23) show significant PPI deficits as compared with control (sham-irradiated) rats (n = 21). (B) Amplitude (in arbitrary units) of acoustic startle responses to the 120 dB auditory stimuli without prepulse in both groups was not different. Data are given as means±SEM. *p<0.05, **p<0.01 as compared with control (sham-irradiated) rats.

**Figure 6:**
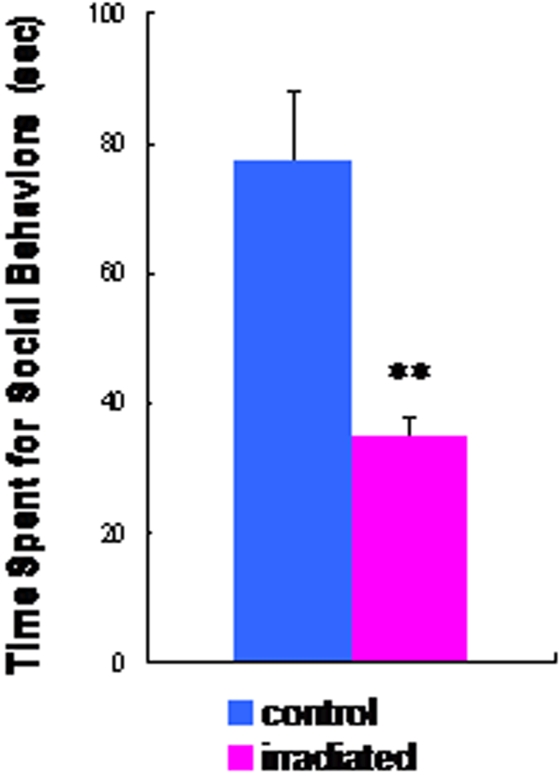
Social withdrawal in the irradiated adult rats. Social interaction test: Total time (sec) spent in social behaviors for 10 min in the irradiated rats (n = 6) was significantly lower than that of control rats (n = 6). Data are given as means±SEM. **p<0.01 as compared with control (sham-irradiated) rats.

**Figure 7:**
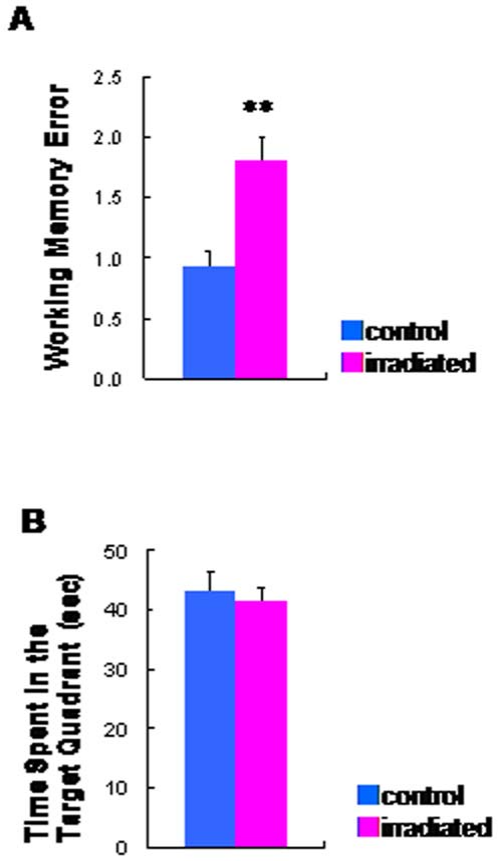
Cognitive impairments in the irradiated adult rats. (A) Spatial working memory in the eight-arm radial maze with 30-sec delay. Total number of revisits to arms from which pellets had already been taken, (i.e., working memory error) is represented as mean±SEM. Irradiated rats (n = 17) showed a higher number of working memory errors than control (sham-irradiated) rats (n = 15). (B): In the probe trials of the Morris water maze, spatial reference memory was intact in the irradiated rats (n = 12). Data are given as means±SEM. **p<0.01 as compared with control (sham-irradiated) rats (n = 12).

In the sensorimotor gating test, two-way ANOVA revealed a significant effect [*F* (1, 42) = 47.1, p<0.001] of irradiation exposure on prepulse inhibition (PPI) (**Figure 5A**), while acoustic response amplitude in the two groups was not different (**Figure 5B**). PPI deficits in irradiated rats were shown at each level of prepulse intensity (72, 76, and 84 dB) (**Figure 5A**). In the social interaction test, the time spent in social behavior in the irradiated rats was significantly (t = 3.73, df = 10, p = 0.004) lower than that that in the control rats (**Figure 6**). In the eight-arm radial maze test, the number of working memory errors in the irradiated rats was significantly (t = −3.63, df = 27.3, p = 0.001) higher than that of control rats (**Figure 7A**). In contrast, the two groups' times in the probe test of a Morris water maze as an index of spatial reference memory were not different (**Figure 7B**).

**Figure 8:**
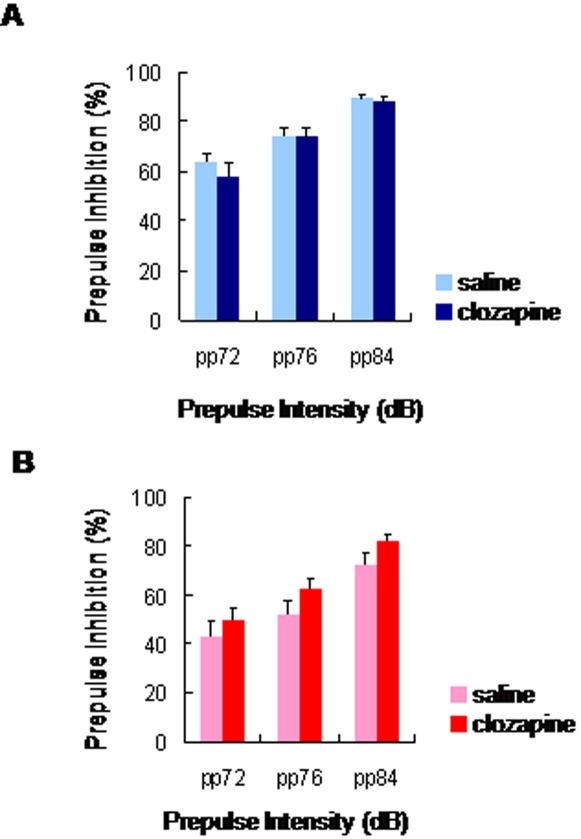
Effects of chronic clozapine administration on PPI deficits. (A) Control (sham-irradiated) rats: Chronic administration of clozapine (5 mg/kg/day for 3 weeks, i.p.) did not alter PPI deficits in the control rats (clozapine: n = 6; vehicle: n = 6). (B) Irradiated rats: Chronic administration of clozapine (5 mg/kg/day for 3 weeks, i.p.) significantly did not alter PPI deficits in the irradiated rats (clozapine: n = 6; vehicle: n = 6). Data are given as means±SEM.

We examined whether the antipsychotic drug clozapine could improve the reduction of neurogenesis and PPI deficits in irradiated rats. Subsequent chronic administration of clozapine (5 mg/kg/day for 3 weeks) did not alter the reduction of neurogenesis in the irradiated (data not shown). Furthermore, subsequent chronic administration of clozapine (5 mg/kg/day for 3 weeks) did not alter PPI in control rats (**Figure 8A**). However, we found that chronic administration of clozapine (5 mg/kg/day for 3 weeks) slightly improved PPI deficits in irradiated rats although a statistical analysis was not significant (**Figure 8B**).

## Discussion

The major findings of the present study are that fractionated ionizing irradiation to the adult male rat brain causes schizophrenia-relevant abnormal behaviors (e.g., methamphetamine-induced hyperactivity, sensory motor gating deficits, social interaction deficits, and working memory deficits) at three months after the irradiation. To the best of our knowledge, this is the first report demonstrating an animal model of schizophrenia by irradiation at adulthood. Although the irradiated adult rats may show essential features (positive and negative symptoms as well as cognitive deficits) relevant to schizophrenia, the pathophysiological mechanism underlying these behavioral changes remains unclear. A recent study using postmortem brain samples demonstrated that proliferation of hippocampal neural stem sells was significantly reduced in patients with schizophrenia, but not unipolar depression [16], suggesting that reduced neural stem cell proliferation may contribute to the pathogenesis of schizophrenia. Moreover, it has been reported that the reduction of cell proliferation in the SGZ after repeated administration of the NMDA receptor antagonist phencyclidine (PCP) may occur in tandem with PCP-induced behavioral changes in rats [22]. In this regard, it is likely that reduction of adult neurogenesis by irradiation may be involved in the schizophrenia-like behavioral abnormalities in rats. Recently, the association between neurogenesis dysfunction and schizophrenia has been also demonstrated [23].

Monje et al. [11] observed that irradiation of the brains of adult rats produced neural progenitor cell dysfunction within the neurogenic zones of the hippocampus, regions plausibly implicated in cognitive deficits. Furthermore, it has been suggested that irradiation-induced cognitive deficits in animals may be associated with a decrease in hippocampal proliferation and a decrease in adult neurogenesis [9]–[15]. In the eight-arm radial maze test, irradiated rats showed a deficit in working memory, which is also shown in schizophrenic patients [24]. It has been suggested that adult neurogenesis may serve an important role in hippocampal-dependent memory processes [25], [26]. First, exposure to an enriched environment or increased physical activity leads to increased hippocampal neurogenesis and improved spatial memory [26]–[28]. Second, the comprehensive loss of hippocampal-dependent memory function in old age is related to decreased neurogenesis [29]. Taken together, it seems that cognitive impairment in irradiated rats may be due to reduction of hippocampal neurogenesis.

In this study, we found that methamphetamine-induced hyperactivity was significantly enhanced in the irradiated rats, suggesting hyperdopaminergic activity. The precise mechanisms underlying the hyperdopaminergic states in irradiated rats could not be explained, as we found no alteration of dopamine or its major metabolite DOPAC in the irradiated rat brains. Since mesolimbic dopaminergic neurons innervate the SGZ of the dentate gyrus [30], the dopaminergic activities of these neurons may be involved in the regulation of hippocampal neurogenesis. Furthermore, we previously reported that cell destruction of dentate granules by intrahippocampal injection of colchicine enhanced methamphetamine-induced hyperactivity in rats [31], suggesting that dentate granule cells may regulate methamphetamine-induced behavioral changes. Taken together, the evidence suggests that the decrease in hippocampal neurogenesis by irradiation may, in part, be implicated in the hyperdopaminergic activity of irradiated rats although the cumulative numbers of granule cells in the granule layer were not altered in irradiated rats.

Accumulating evidence suggests that hypofunction of the NMDA receptors may play a role in the pathophysiology of schizophrenia [32]–[34]. However, we did not find any alteration in dizocilpine-induced hyperactivity and levels of amino acids related to NMDA receptor neurotransmission in irradiated rat brains. Therefore, it is unlikely that alteration in the NMDA receptors is involved in the behavioral abnormalities in irradiated rats, although further studies are necessary.

The idea that antipsychotic drugs may increase neurogenesis in the rat hippocampus has not been consistently supported [17]. In this study, we found that chronic administration of clozapine (5 mg/kg/day for 3 weeks) did not alter the reduction of neurogenesis in the irradiated and control rats. In addition, we found that chronic administration of clozapine (5 mg/kg/day for 3 weeks) significantly did not improve PPI deficits in irradiated rats although a slight improvement by clozapine was shown. Therefore, it is likely that the inefficiency of clozapine treatment on PPI deficits in irradiated rats may be dependent upon the reduction of adult neurogenesis, although a further study will be necessary.

The total numbers of BrdU-positive cells in both SVZ and SGZ were significantly lower than those of sham-irradiated rats three months after fractionated irradiation. The static BrdU-positive cell count may reflect neurogenesis and/or survival of the recent born cells. Monje et al. [10] have demonstrated that normal number of neural progenitors was surviving two months after radiation exposure although proliferative activity was reduced. They also have shown that the neural stem/precursor cells isolated from irradiated hippocampi failed to expand only 2–3 passages. These findings suggest that the reduction of proliferating cells in the present study might be due to ablated neurogenesis without reduction of cell survival. Actually, it is reported that the decline of proliferating cells lasted at 15 months after irradiation [35].

In the present study, we have regarded the neurogenesis dysfunction as a possible mechanism underlying the radiation induced abnormal behaviors associated with schizophrenia, based on the findings suggesting the link between neurogenesis dysfunction and schizophrenia. However, it has been reported that irradiation also induces apoptosis [9], neuroinflammation [11], and loss of oligodendrocyte precursor [35]. Further detailed investigation is required to discriminate the involvement of these factors on irradiation induced abnormal behavior relevant to schizophrenia. Further studies on the optimization of radiation dose, phenotypic alteration by the exposure age, and sex differences are also needed.

In conclusion, the present findings suggest that irradiation in adulthood might provide a new animal model of schizophrenia. Further understanding the molecular and cellular mechanisms underlying the behavioral abnormalities in irradiated rats would contribute to the pathophysiology of schizophrenia.
